# High circulating MIF levels indicate the association with atypical antipsychotic-induced adverse metabolic effects

**DOI:** 10.1038/s41398-024-02934-8

**Published:** 2024-05-27

**Authors:** Xi Chen, Pingyi Gao, Yadan Qi, Zezhi Li, Hongna Huang, Yuan Shi, Lijun Wang, Donghong Cui, Dake Qi

**Affiliations:** 1 Shanghai Mental Health Center, Shanghai Jiao Tong University School of Medicine, Shanghai, China; 2 College of Pharmacy, Rady Faculty of Health Sciences, University of Manitoba, Winnipeg, MB Canada; 3 Shanghai Key Laboratory of Psychotic Disorders, Shanghai, China; 4 Department of Psychiatry, the Affiliated Brain Hospital of Guangzhou Medical University, Guangzhou, China; 5 Guangdong Engineering Technology Research Center for Translational Medicine of Mental Disorders, Guangzhou, China; 6 Brain Science and Technology Research Center, Shanghai Jiao Tong University, Shanghai, China

**Keywords:** Biomarkers, Diseases

## Abstract

Atypical antipsychotics (AAPs) are primary medications for schizophrenia (SZ). However, their use is frequently associated with the development of adverse metabolic effects, and the mechanisms behind these negative effects remain inadequately elucidated. To investigate the role of macrophage migration inhibitory factor (MIF) in regulating antipsychotic-induced metabolic abnormalities, between 2017 and 2020, a cross-sectional study was conducted, involving 142 healthy individuals and 388 SZ patients undergoing treatment with either typical antipsychotic (TAP) or AAP medications. Symptoms of SZ patients were evaluated using the Positive and Negative Syndrome Scale (PANSS), and measurements of metabolic indices and plasma MIF levels were performed on all individuals. A significant increase in plasma MIF levels was observed in groups receiving five major AAP monotherapies in comparison to healthy controls (all *p* < 0.0001). There was no such increase shown in the group receiving TAP treatment (*p* > 0.05). Elevated plasma MIF levels displayed a notable correlation with insulin resistance (β = 0.024, *p* = 0.020), as well as with the levels of triglycerides (β = 0.019, *p* = 0.001) and total cholesterol (β = 0.012, *p* = 0.038) in the groups receiving AAPs. However, while the TAP group also displayed certain metabolic dysfunction compared to healthy controls, no significant association was evident with plasma MIF levels (all *p* > 0.05). In conclusion, plasma MIF levels exhibit a distinctive correlation with metabolic abnormalities triggered by AAPs. Hence, there is potential for further development of MIF as a distinctive marker for monitoring adverse metabolic effects induced by AAPs in clinical settings.

## Introduction

Atypical antipsychotic (AAP) therapy frequently leads to adverse metabolic effects [1–3], which were previously attributed to the impact of AAPs on various neural receptors, including dopamine, adrenergic, serotonin, histamine, and muscarinic receptors [4, 5]. However, common metabolic abnormalities are intricately linked to adipose tissue dysfunction, leading to the altered release of “adipokines”, which are adipose-derived proteins serving as key contributors to the onset of insulin resistance, obesity, and hyperlipidemia. Therefore, the levels of circulating adipokines represent important indices of metabolic dysfunction and need to be fully elucidated.

The classical inflammatory cytokine, macrophage migration inhibitory factor (MIF) [6], is also recognized as an adipokine [7]. Obese individuals often display higher plasma MIF levels compared to their lean counterparts [8], suggesting a strong correlation between MIF and obesity. Plasma MIF levels demonstrate a positive correlation with insulin resistance in patients with type 2 diabetes (T2D) [9, 10]. Additionally, they also play a contributory role in the development of non-inflammatory insulin resistance in obese animal models [11]. Furthermore, increased circulating MIF levels play a role in inducing hypertriglyceridemia by downregulating lipoprotein lipase (LPL) in adipose tissue [12]. Therefore, elevated plasma MIF levels serve as a significant indicator for monitoring the progression of metabolic dysfunction.

Previous studies have established a potential relationship between MIF and severe mental disorders, such as schizophrenia (SZ) and bipolar disorders (BD). The *MIF* gene polymorphism has been linked to the onset and risk of SZ [13], as well as to attempted suicide in BD [14]. In Japanese patients with SZ, serum MIF level is higher compared to healthy controls, and it may be correlated with antipsychotic therapy [15, 16]. We previously found that administering olanzapine (OLZ), a common AAP, as monotherapy over two months led to increased circulating MIF levels in first-episode SZ patients, and this rise was positively associated with elevated body weight gain, insulin resistance, and hyperlipidemia [17]. Our animal study revealed that the increase in circulating MIF levels primarily comes from MIF expression and release in adipocytes following OLZ administration [17] Therefore, *Mif* knockout mice were able to mitigate OLZ-induced metabolic dysfunction [17]. This collective evidence suggests that MIF may serve as a crucial regulator for adverse metabolic effects induced by OLZ.

It should be noted that adverse metabolic effects are a common occurrence across various AAP therapies. The question of whether MIF plays a role in other AAP-induced metabolic abnormalities and whether it could serve as a unique marker for AAP-induced adverse metabolic effects remains to be elucidated. Thus, our current study enrolled healthy controls and patients with SZ who were receiving various antipsychotic monotherapies, including one typical and five atypical antipsychotics, respectively. We were investigating the correlation between MIF levels and metabolic side effects associated with antipsychotic treatments.

## Materials and Methods

### Study design and participants

The cross-sectional investigation comprised Han Chinese individuals for both healthy controls and SZ patients. A total of 142 healthy subjects were recruited locally through advertisements. Chronic SZ patients were recruited from various healthcare centers, including the Shanghai Mental Health Center, Shanghai Civil Affairs First Mental Health Center, Cixi Mental Health Center, Quzhou Third People’s Hospital, and Huzhou Third People’s Hospital during the period spanning from January 2017 to January 2020. The Inclusion criteria for SZ patients included: (1) age eligibility was set between 18 and 65 years; (2) SZ diagnosis was made according to the Diagnostic and Statistical Manual of Mental Disorders Fifth Edition (DSM-5) by at least two separate psychiatrists*;* (3) SZ patients must have received at least six months of antipsychotic treatment. The exclusion criteria for SZ patients included (1) the presence of cerebral vascular disease, central nervous system diseases, severe physical diseases, and substance abuse/dependence; (2) genetic syndromes linked to obesity or other chronic conditions were grounds for exclusion; (3) individuals using medications for the treatment of insulin resistance or T2D were ineligible; or (4) pregnant or lactating women were also excluded. A total of 388 SZ patients met the aforementioned criteria and were ultimately included in the statistical analysis.

The authors affirm that all procedures involved in this study adhere to the ethical standards set by the relevant national and institutional committees on human experimentation and comply with the Helsinki Declaration of 1975, as revised in 2008. All procedures involving human subjects were approved by the Institutional Review Boards of the Shanghai Mental Health Center (2019-35 R). All potential participants were provided with comprehensive written information about the project.

#### Sample size calculation

Sample size calculation was performed using GPower v.3.1 9.7. The evaluation employed the analysis of variance (ANOVA) statistical method for 7 groups, expecting an effect size of 0.4 [18], with an alpha error of 0.05 and a power of 80%. Given equal sample sizes across the 7 groups, the minimum required sample size per group was determined to be 14 cases. Considering a dropout rate of 20%, each of the 7 groups was expanded to include 18 cases, resulting in a total sample size of 126 cases. This ensures the accuracy and scientific rigor of the study findings.

### Collection of data and blood samples

All measurements were performed by trained research nurses. All participants underwent waist circumference (WC), and hip circumference (HC) measurements. The Waist-hip ratio (WHR) was calculated by dividing WC by HC. Blood samples were collected between 7:00 and 9:00 AM following a fasting period of 12–14 h. These collected blood serum samples were used to obtain the insulin, fasting glucose (Fpg), hemoglobin A1c (HbA1c), total cholesterol (TC), low-density lipoprotein (LDL), high-density lipoprotein (HDL), triglycerides (TG), apolipoprotein A1 (ApoA1), apolipoprotein B (ApoB), and the concentration of MIF. These metabolic parameters were detected by the Department of Clinical Laboratory, Shanghai Mental Health Center. The levels of MIF were determined by ELISA using a commercially available kit (SEA698Hu, USCN Life, Wuhan, China), with a standard curve generated using recombinant human MIF. The mean intra- and inter-assay coefficient of variation values were 4.6% and 7.8%, respectively. The lower limit of detection was 0.122 ng/ml. The HOMA-IR index was calculated using the formula: HOMA-IR = [Insulin μU/mL] x [Glucose mM]/22.5.

### Clinical assessment

Clinical interviews with the participants were conducted in a structured format. Trained clinicians with experience in the protocol conducted these interviews. Socio-demographic information and clinical characteristics, including gender, age, education, age of onset, duration of illness, antipsychotic use, and other medications, were collected using a questionnaire. Clinical symptoms were assessed using the Positive and Negative Syndrome Scale (PANSS), and the inter-rater correlation coefficient of assessments exceeded 0.8.

### Statistical analysis

Statistical analyses were conducted using GraphPad Prism version 8.0 (GraphPad Software, San Diego, USA) and SPSS 16.0 (SPSS, Chicago, USA). Data underwent normal distribution testing for continuous variables using the Kolmogorov-Smirnov one-sample test. Continuous variables were presented as means with standard deviations or medians with interquartile ranges, depending on their distribution. Parametrically distributed variables were analyzed using ANOVA, while nonparametric variables were assessed using the Kruskal-Wallis test. Gender variables were analyzed with the chi-square (χ2) test.

To compare the differences in plasma MIF levels and metabolic markers between groups, we conducted a multifactor ANOVA. Post-hoc analyses (if applicable) were further conducted to explore pairwise differences between groups. Multiple linear regression was employed to determine correlations between MIF and other indices. In all these analyses, we incorporated relevant variables for adjustment, including age, gender, WHR, and PANSS scores. The hypothesis test was two-sided with a 5% level of statistical significance, and a 95% confidence interval was applied.

## Results

### Subject background characteristics

Among the SZ patients, 32 received monotherapy with chlorpromazine (CPZ), a typical antipsychotic (TAP), while the remaining patients obtained various AAP monotherapies over the past six months. Specifically, 114 patients were treated with OLZ, 77 patients received CLZ, and 41, 93, and 31 patients were treated with aripiprazole (APZ), risperidone (RIS), and quetiapine (QTP), respectively. A comprehensive overview of the baseline background characteristics for each group is shown in Table 1. The analysis revealed significant differences among the groups in terms of sex, age, education, and duration of illness (*p* < 0.05), while no significant difference was observed regarding age of onset (*p* = 0.838). Although the total PANSS score varied among different patient groups, the negative score did not exhibit statistical significance (*p* = 0.789).

**Table 1:** Demographic information and clinical characteristics of healthy controls and patients with SZ.

Variables	HC (*n* = 142)	CPZ (*n* = 32)	OLZ (*n* = 114)	CLZ (*n* = 77)	APZ (*n* = 41)	RIS (*n* = 93)	QTP (*n* = 31)	Test Statistic	*p v*alue
Age^a^, yrs	35.37 (12.64)	55.13 (14.63)	36.29 (13.91)	47.73 (15.30)	39.44 (17.45)	36.79 (15.26)	41.74 (14.63)	13.990	**0.000**
Sex^b^	66 (46.5) / 76 (53.5)	9 (28.1) /23 (71.9)	41 (36.0) / 73 (64.0)	20 (26.0) / 57 (74.0)	20 (48.8) / 21 (51.2)	37 (39.8) / 56 (60.2)	11 (35.5) / 20 (64.5)	12.700	**0.048**
Education^a^, yrs	5.06 (1.37)	3.66 (0.83)	3.97 (0.98)	3.43 (0.93)	3.87 (0.86)	4.04 (0.91)	3.50 (0.95)	26.066	**0.000**
Age of onset^a^, yrs		29.13 (12.39)	27.81 (11.76)	27.13 (9.92)	27.10 (8.09)	26.26 (10.67)	29.17 (8.77)	0.414	0.838
Duration of illness^a^, months		269.54 (193.92)	121.85 (154.39)	209.82 (194.85)	216.07 (212.36)	171.50 (162.57)	163.87 (148.10)	3.212	**0.008**
PANSS score									
Positive^c^		14.00 (12.00)	20.50 (15.00)	15.00 (13.00)	19.00 (13.00)	19.00 (15.50)	19.00 (9.00)	19.774	**0.001**
Negative^c^		14.00 (13.75)	16.00 (11.50)	16.00 (14.00)	19.00 (10.5)	16.00 (10.00)	16.00 (12.00)	2.416	0.789
General^c^		28.50 (12.75)	38.00 (21.5)	33.00 (21.25)	37.00 (32.75)	34.00 (26.00)	38.00 (14.00)	20.344	**0.001**
Total^c^		62.50 (27.25)	79.00 (41.50)	64.00 (38.75)	77.50 (42.5)	72.00 (42.00)	79.00 (33.50)	17.915	**0.003**

^a^Mean (SD); ^b^Female n (%)/male n (%); ^c^Median (IQR). Test Statistic: χ² for the Chi-square test, H for the Kruskal-Wallis test, F for One-way ANOVA

### AAPs specifically upregulate plasma MIF levels in SZ patients

**Fig. 1:**
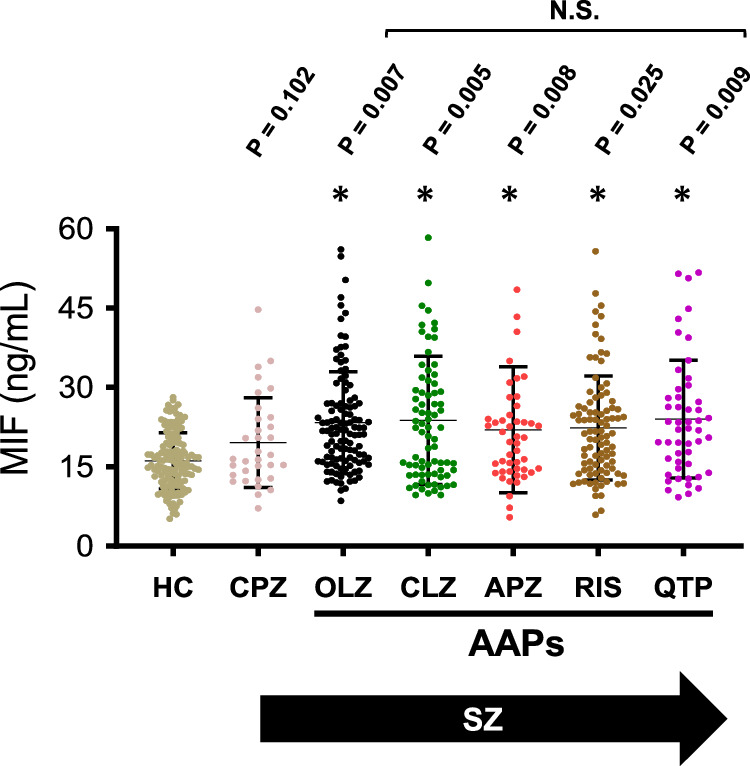
Plasma MIF levels in healthy controls and patients with SZ. Dunnett’s test was used for Post hoc multiple comparisons (to healthy controls, HC). All *P* values were adjusted for age, gender, and WHR in multi-factor ANOVA. * *P* value < 0.05. N.S. represents no significant difference.

We examined plasma MIF levels in both healthy controls and SZ patients undergoing various antipsychotic monotherapies (Fig. 1). CPZ did not change plasma MIF levels (*p* = 0.102). However, all AAPs upregulate plasma MIF levels among SZ patients when compared to both healthy controls and patients receiving TAP treatment (*p* < 0.05). There were no significant differences in the degree of MIF elevation within the AAP groups.

### MIF and waist-hip ratio (WHR) following AAP therapies

The waist-hip ratio (WHR) serves as a vital indicator for assessing abdominal obesity. WHR was significantly increased among all patients receiving AAPs compared to the healthy controls (Table S1). Furthermore, given the established association between MIF and the development of abdominal adiposity [19], our multiple linear regression analysis revealed a positive correlation between plasma MIF levels and WHR (β = 0.001, *p* = 0.011).

### MIF and AAP-induced insulin resistance

AAPs resulted in elevated HOMA-IR scores compared to those observed in healthy controls (Table S2). Our correlation analysis within the patient groups receiving AAPs (Table 2) unveiled a positive association between plasma MIF concentration and both HOMA-IR scores (β = 0.024, *p* = 0.020) and insulin levels (β = 0.903, *p* = 0.001). However, no statistically significant associations were found between MIF and Fpg (*p* = 0.977) or HbA1c (*p* = 0.725).

**Table 2:** Association of plasma MIF concentration with glucose metabolism indexes in patients with SZ (OLZ, CLZ, APZ, RIS, QTP).

Variables	β	*p* value
HOMA-IR	0.024	**0.020**
Insulin	0.903	**0.001**
Fasting glucose (Fpg)	0.001	0.977
HbA1c	0.003	0.752

All *P* values were adjusted for age, gender, and PANSS.

### MIF is correlated with plasma triglyceride levels

Evaluation of lipid metabolism indices, including TC, LDL, HDL, TG, ApoA1, and ApoB, was conducted across seven groups (Table S3). The administration of AAPs did not exert an influence on TC levels but significantly decreased HDL levels in all groups. Specifically, OLZ and CLZ elevated LDL levels, while CLZ and APZ increased TG levels in comparison to healthy individuals (Table S3). Moreover, CLZ treatment led to increased levels of ApoB. These findings suggest that CLZ therapy induces more pronounced hyperlipidemia when compared to other AAPs. A positive correlation was identified solely between plasma MIF concentration with TC (β = 0.012, *p* = 0.038) and TG values (β = 0.019, *p* = 0.001) (Table 3).

**Table 3:** Association of plasma MIF concentration with lipid metabolism indexes in patients with SZ (OLZ, CLZ, APZ, RIS, QTP).

Variables	β	p-value
TC	0.012	**0.038**
LDL	0.008	0.081
HDL	0.001	0.694
TG	0.019	**0.001**
ApoA1	-0.001	0.663
ApoB	0.002	0.707

All *P* values were adjusted for age, gender, and PANSS.

### MIF and TAP-induced metabolic alterations

Patients receiving the TAP treatment of CPZ did not exhibit alterations in their plasma MIF levels. However, they did experience elevated HOMA-IR, Fpg, and HbA1c levels when compared to healthy controls (Table S2). In contrast to the group receiving AAP treatment, these CPZ-treated patients did not manifest hyperinsulinemia, and correlation analysis also revealed no significant relationship between MIF and glucose metabolism indices (all *p* > 0.05, Table S4). Furthermore, CPZ regulated lipid metabolism, characterized by decreased HDL levels (*p* < 0.001) and increased ApoA1 (*p* < 0.001) and ApoB levels (*p* = 0.014) (Table S3). Importantly, there was also no observed correlation between lipid metabolism indices and plasma MIF (all *p* > 0.05, Table S4).

## Discussion

While traditionally categorized as a cytokine, MIF has garnered increasing attention for its pivotal role in driving the progression of metabolic dysfunction [20]. We revealed an important association between the usage of AAPs and the upregulation of MIF levels in SZ patients. The presence of metabolic dysfunction is accompanied by an increase in circulating MIF levels in SZ patients undergoing AAP treatments. Notably, the heightened MIF levels are closely linked to insulin resistance, with a primary reliance on increased blood insulin levels rather than glucose. Furthermore, plasma MIF levels are associated with plasma TC and TG levels following AAP treatments. In contrast, despite there is the presence of adverse metabolic effects induced by TAP treatment, the plasma MIF level remains unchanged. Therefore, we propose that MIF could serve as a distinctive marker to monitor adverse metabolic effects induced by AAPs in clinical settings.

Adipokines are cell-signaling molecules derived from adipose tissue, responsible for regulating the body’s metabolic state. Alterations in plasma adipokine levels frequently coincide with AAP-induced metabolic dysfunction, although the precise nature of this relationship remains unclear. SZ patients often exhibit higher levels of leptin compared to healthy controls, and these elevated levels can persist throughout antipsychotic therapy [21, 22]. While OLZ, CLZ, and QTP are known to significantly increase leptin levels, it remains uncertain whether their influence on leptin levels is direct or mediated indirectly through effects on obesity and subsequent leptin resistance [21]. AAPs have also been observed to decrease adiponectin levels [23] and promote ghrelin secretion [24]. However, it remains uncertain whether these alterations are dependent upon adiposity and BMI. Unlike leptin, adiponectin, and ghrelin, MIF is directly upregulated in mouse adipose tissue and adipocytes by OLZ [17]. The upregulation of adipose MIF leads to elevated circulating MIF levels, which in turn contribute to the development of metabolic abnormalities [17]. Indeed, OLZ monotherapy in SZ patients for two months also elevated plasma MIF levels, thereby contributing to insulin resistance, obesity, and hyperlipidemia [17]. Our findings regarding the interesting correlation between MIF and OLZ have prompted a hypothesis suggesting that plasma MIF levels could potentially influence the metabolic side effects induced by all major AAPs. However, this hypothesis remained unexplored until our current investigation. Our current study explores the involvement of MIF in the development of obesity, insulin resistance, and hyperlipidemia caused by other AAPs, including CLZ, APZ, RIS, and QTP, which are commonly prescribed in clinical settings.

The chronic therapy of AAPs is commonly associated with the development of obesity. In animal models, MIF plays a crucial role in regulating lipid storage in adipocytes, which leads to adipocyte hypertrophy and increased adiposity following high-fat diet feeding [25]. Our present study indicates a positive correlation between plasma MIF levels and AAP-induced enlargement of WHR, suggesting a potential role of MIF in accelerating the development of AAP-induced central obesity. Previously, our animal study also showed a novel molecular mechanism of MIF in modulating hyperphagia through an AMPK/neuropeptide, AgRP signaling pathway [17]. The extent to which elevated peripheral MIF can access the hypothalamus and regulate appetite in SZ patients undergoing AAP treatment remains largely unknown.

Plasma MIF levels are frequently associated with insulin resistance [9, 10]. Our current study suggests a positive association between elevated plasma MIF levels and hyperinsulinemia following AAP treatment. OLZ amplified *Mif* expression in both the hypothalamus and adipose tissue, with the latter significantly contributing to the increase in plasma MIF levels in animal models [17]. Subsequently, the increased levels of circulating MIF were associated with the presence of insulin resistance within adipose tissue, facilitated by the inhibition of tyrosine phosphorylation of insulin receptor substrate-1 (IRS-1) [26] and the disturbance of the signaling pathway responsible for glucose metabolism [26]. Interestingly, in our present study, we did not conclude any correlation between MIF and plasma glucose or HbA1c levels, despite a previous study suggesting a stepwise increase in serum MIF associated with the progression from impaired glucose tolerance (IGT) to type 2 diabetes (T2D) [27]. Two potential rationales may explain the phenomenon. First, the progression of insulin resistance stages has not reached a point where significant changes in glucose levels occur. Second, MIF has been reported to stimulate insulin release from beta cells [28]. It is currently unknown whether this effect contributes to the balance of dysfunctional glucose metabolism.

Moreover, our research revealed the presence of lipid abnormalities induced by AAPs, with high plasma MIF levels showing a specific correlation with plasma TG and TC values. Our previous longitudinal study suggested a positive correlation between changes in plasma TG and alterations in plasma MIF levels following two months of OLZ monotherapy [17]. Recent findings also indicated that MIF directly inhibits the expression and activity of lipoprotein lipase (LPL) in mouse adipose tissue [12], thereby impairing LPL’s ability to break down plasma TG, resulting in hypertriglyceridemia. Further investigation is needed to determine whether LPL is involved in MIF-regulated AAP-induced hypertriglyceridemia in patients with SZ. This can be achieved by conducting a study where LPL is released using heparin, as described in previous studies [29].

Interestingly, elevated plasma MIF levels exhibit a positive correlation with TC rather than LDL. TC encompasses various cholesterol types carried by lipoprotein particles, such as LDL, HDL, intermediate-density lipoproteins (IDL), and very low-density lipoprotein (VLDL) [30]. While LDL accounts for 60% of total cholesterol in the body [31], other types of cholesterol also play important roles in metabolism. Previous studies have suggested that diabetic patients often exhibit low LDL/high remnant cholesterol (RC) profiles rather than high LDL/low RC profiles [32], with this phenomenon being more pronounced in female patients [32]. Thus, our discovery regarding the association between MIF and TC may imply a potential role of MIF in regulating non-LDL cholesterols.

While the prevailing perspective suggests that TAPs are less likely to induce metabolic abnormalities compared to AAPs, our research, along with findings from other research groups [33, 34], have demonstrated metabolic dysfunction induced by TAPs. In a cohort of SZ patients receiving CPZ monotherapy for over a decade, the prevalence of metabolic syndromes was found to be 31% [35]. In our current research, we did observe the occurrence of metabolic dysfunction in the CPZ group, characterized by insulin resistance and alterations in plasma levels of HDL, ApoA1, and ApoB levels. CPZ-induced insulin resistance appeared to be associated with increased plasma glucose levels rather than insulin levels, in contrast to the insulin resistance induced by MIF. In addition, CPZ did not elevate plasma MIF levels compared to those of healthy controls, although the sample size is small. The unchanged MIF levels in the CPZ group suggest that TAP-induced metabolic dysfunction is probably more predominantly associated with an MIF-independent mechanism. It should be noted that MIF has the capability to metabolize dopaminechrome into 3,4-dihydroxyphenylacetone [36], thereby alleviating extrapyramidal symptoms. Typically, the oxidation of dopamine leads to the formation of toxic dopaminechrome, a precursor of neuromelanin, which accumulates in neurons and astrocytes, resulting in extrapyramidal symptoms [37]. Thus, the MIF-independent mechanism in TAPs could partially explain their extrapyramidal side effects.

Although our findings present pioneering evidence supporting the link between plasma MIF levels and AAPs-induced metabolic disturbances, our study design does come with several limitations. Firstly, our research design was cross-sectional, which restricted our ability to establish the alterations between MIF levels and all the metabolic indexes before and after the monotherapy. The other limitation of our study was the medication dose was not factored into our statistical analysis, potentially influencing metabolic measurements. Future studies employing longitudinal designs and incorporating baseline measurements would provide a more comprehensive understanding of the relationship between MIF and AAP-induced metabolic dysfunctions. Furthermore, all participants in our study were Han Chinese. There are 56 ethnic groups in China, with Han accounting for 91.51% and various ethnic minorities accounting for 8.49% [38]. Given that Han Chinese make up the majority, our sample adequately represents the Chinese and Eastern Asian populations. However, to validate and extend our findings, further exploration of diverse populations around the world is necessary in the future.

In conclusion, we have identified MIF as a potential clinical marker for recognizing patients at risk of developing metabolic dysfunction induced by AAPs. Our study suggests that patients with elevated MIF levels may require more frequent monitoring for the detection of early signs of AAP-induced metabolic dysfunction. In addition, exploring interventions targeting MIF or its associated signaling pathways may be a possible strategy to prevent AAP-induced adverse metabolic effects and improve their therapeutic effects in clinical settings.

### Supplementary information

Supplemental Material

## Data Availability

All original data supporting the current study are available upon request.
