# Supplementary material for: High circulating MIF levels indicate the association with atypical antipsychotic-induced adverse metabolic effects
Source: Transl Psychiatry. 2024 May 27;14:210. doi: 10.1038/s41398-024-02934-8 (PMC11130196; doi:10.1038/s41398-024-02934-8)
Supplement: Supplementary file 1 — Supplemental Material [file 41398_2024_2934_MOESM1_ESM.docx]

Table S1. The association of plasma MIF concentration with WHR in healthy controls and patients with SZ (OLZ, CLZ, APZ, RIS, QTP).

| WHR | mean ± SD | p-value |
| --- | --- | --- |
| HC | 0.86 ± 0.09 |  |
| OLZ | 0.90 ± 0.12 | **0.010 ^a^** |
| CLZ | 0.92 ± 0.07 | **0.000 ^a^** |
| APZ | 0.92 ± 0.07 | **0.004 ^a^** |
| RIS | 0.90 ± 0.10 | **0.006 ^a^** |
| QTP | 0.92 ± 0.07 | **0.011 ^a^** |
| F | 2.258 | **0.041 ^b^** |
| β | 0.001 | **0.011 ^c^** |

^a^ P values of Dunnett's test which were adjusted for age and gender. ^b^ P values of multi-factor ANOVA which were adjusted for age and gender. ^c^ P values of multiple linear regression which were adjusted for age, gender and PANSS.

Table S2. Glucose metabolism indexes of healthy controls and patients with SZ.

| Variables | | HC  (n=142) | CPZ  (n=32) | OLZ  (n=114) | CLZ  (n=77) | APZ  (n=41) | RIS  (n=93) | QTP  (n=31) | F | p-Value ^b^ |
| --- | --- | --- | --- | --- | --- | --- | --- | --- | --- | --- |
| HOMA-IR | mean (SD) | 0.91 (0.47) | 1.72 (1.46) | 2.46 (2.06) | 2.06 (1.51) | 2.59 (1.97) | 2.15 (1.50) | 2.27 (2.10) |  |  |
|  | p-value ^a^ |  | **0.036** | **0.000** | **0.000** | **0.000** | **0.000** | **0.000** | 7.744 | **0.000** |
| insulin | mean (SD), μU/ml | 26.43 (17.27) | 42.70 (51.73) | 70.40 (58.93) | 50.95 (44.07) | 72.18 (53.23) | 60.28 (39.98) | 62.54 (50.76) |  |  |
|  | p-value ^a^ |  | 0.289 | **0.000** | **0.001** | **0.000** | **0.000** | **0.000** | 6.812 | **0.000** |
| fpg | mean (SD), μU/ml | 4.65 (0.62) | 5.70 (1.45) | 5.25 (1.23) | 5.80 (1.76) | 5.69 (1.76) | 5.27 (1.10) | 5.16 (0.76) |  |  |
|  | p-value ^a^ |  | **0.000** | **0.000** | **0.000** | **0.000** | **0.001** | 0.154 | 5.168 | **0.000** |
| A1c | mean (SD), % | 5.35 (0.36) | 5.82 (0.97) | 5.53 (1.73) | 5.92 (1.33) | 6.00 (1.91) | 5.48 (1.02) | 5.37 (0.48) |  |  |
|  | p-value ^a^ |  | **0.043** | 0.440 | **0.000** | **0.000** | 0.858 | 1.000 | 2.032 | 0.063 |

^a^ P values of Dunnett's test. ^b^ P values of multi-factor ANOVA

All P values were adjusted for age and gender.

Table S3. Lipid metabolism indexes of healthy controls and patients with SZ.

| Variables | | HC  (n=142) | CPZ  (n=32) | OLZ  (n=114) | CLZ  (n=77) | APZ  (n=41) | RIS  (n=93) | QTP  (n=31) | F | p-Value ^b^ | |
| --- | --- | --- | --- | --- | --- | --- | --- | --- | --- | --- | --- |
| TC | mean (SD), mmol/L | 4.22 (0.74) | 4.57 (1.05) | 4.43 (1.09) | 4.37 (1.08) | 4.29 (0.85) | 4.25 (0.91) | 4.69 (0.93) |  |  |  |
|  | p-value ^a^ |  | 0.257 | 0.334 | 0.788 | 0.998 | 1.000 | 0.064 | 1.928 | 0.078 |  |
| LDL | mean (SD), mmol/L | 2.43 (0.80) | 2.81 (0.95) | 2.69 (0.95) | 2.73 (0.78) | 2.65 (0.73) | 2.57 (0.77) | 2.75 (0.88) |  |  |  |
|  | p-value ^a^ |  | 0.058 | **0.036** | **0.026** | 0.448 | 0.636 | 0.163 | 2.409 | **0.029** |  |
| HDL | mean (SD), mmol/L | 1.67 (0.49) | 1.24 (0.39) | 1.29 (0.34) | 1.24 (0.40) | 1.30 (0.39) | 1.25 (0.35) | 1.43 (0.31) |  |  |  |
|  | p-value ^a^ |  | **0.000** | **0.000** | **0.000** | **0.000** | **0.000** | **0.011** | 10.094 | **0.000** |  |
| TG | mean (SD), mmol/L | 1.14 (0.57) | 1.28 (0.66) | 1.22 (0.64) | 1.46 (0.96) | 1.56 (1.27) | 1.16 (0.69) | 1.33 (0.77) |  |  |  |
|  | p-value ^a^ |  | 0.777 | 0.831 | **0.002** | **0.001** | 1.000 | 0.498 | 3.731 | **0.002** |  |
| ApoA1 | mean (SD), g/L | 1.28 (0.19) | 1.54 (1.26) | 1.27 (0.27) | 1.29 (0.39) | 1.30 (0.27) | 1.24 (0.31) | 1.37 (0.32) |  |  |  |
|  | p-value ^a^ |  | **0.000** | 0.997 | 1.000 | 1.000 | 0.802 | 0.520 | 2.809 | **0.012** |  |
| ApoB | mean (SD), g/L | 0.78 (0.24) | 1.33 (1.60) | 0.91 (0.87) | 1.17 (1.65) | 0.81 (0.22) | 0.81 (0.30) | 0.87 (0.23) |  |  |  |
|  | p-value ^a^ |  | **0.014** | 0.650 | **0.005** | 1.000 | 1.000 | 0.990 | 1.211 | 0.302 |  |

^a^ P values of Dunnett's test. ^b^ P values of multi-factor ANOVA

All P values were adjusted for age and gender.

Table S4. Association of plasma MIF concentration with metabolic index in patients with SZ (CPZ).

| Variables | β | p-value |
| --- | --- | --- |
| HOMA-IR | 0.033 | 0.340 |
| Insulin | 0.860 | 0.468 |
| Fasting glucose | -0.002 | 0.953 |
| HbA1c | -0.014 | 0.533 |
| TC | 0.019 | 0.428 |
| LDL | 0.008 | 0.729 |
| HDL | 0.018 | 0.057 |
| TG | 0.005 | 0.727 |
| ApoA1 | -0.046 | 0.176 |
| ApoB | -0.023 | 0.655 |
| WHR | 0.001 | 0.862 |

All P values were adjusted for age, gender and PANSS.
